# Effect of Zebularine in Comparison to and in Combination with Trichostatin A on CIP/KIP Family (*p21Cip1/Waf1/Sdi1*, *p27Kip1*, and *p57Kip2*), *DNMTs* (*DNMT1*, *DNMT3a*, and *DNMT3b*), Class *I HDACs* (*HDACs 1, 2, 3*) and *Class II HDACs *(*HDACs 4, 5, 6*) Gene Expression, Cell Growth Inhibition and Apoptosis Induction in Colon Cancer *LS 174T* Cell Line

**DOI:** 10.31557/APJCP.2020.21.7.2131

**Published:** 2020-07

**Authors:** Masumeh Sanaei, Fraidoon Kavoosi

**Affiliations:** *Research Center for Non-Communicable Diseases, Jahrom University of Medical Sciences, Jahrom, Iran. *

**Keywords:** Zebularine, trichostatin, CIP/KIP family, colon cancer

## Abstract

**Background:**

A pattern of epigenetic modifications and changes, DNA methylation and histone modification, is central to many human cancers. A variety of tumor suppressor genes (TSGs) have been demonstrated to be silenced because of histone deacetylation and DNA hypermethylation in several cancers. Recent in vitro studies have shown that two known mechanisms of epigenetic alteration consisting of methylation and histone deacetylation seem to be the best candidate mechanisms for inactivation of *CIP/KI*P family (*p21Cip1/Waf1/Sdi1*, and *p27Kip1*) in numerous cancers. Numerous investigations have indicated that DNA demethylating and histone deacetylase inhibitors (*HDACIs*) can restore the *CIP/KIP* family gene expression. Previously, we evaluated the effect of trichostatin A (TSA) and 5-aza-2′-deoxycytidine (5-AZA-CdR) on hepatocellular carcinoma (HCC). The present study was designed to investigate the effect of zebularine in comparison to and in combination with trichostatin A on *p21Cip1/Waf1/Sdi1, p27Kip1*, *p57Kip2, DNMT1, DNMT3a *and *DNMT3b, Class I HDACs (HDACs 1, 2, 3) *and* Class II HDACs (HDACs 4, 5, 6)* gene expression, cell growth inhibition and apoptosis induction in colon cancer LS 174T cell line.

**Materials and Methods:**

The colon cancer LS 174T cell line was cultured and treated with zebularine and TSA. To determine cell viability, apoptosis, and the relative expression level of the genes, MTT assay, cell apoptosis assay, and qRT-PCR were done respectively.

**Results:**

Both compounds significantly inhibited cell growth, and induced apoptosis. Furthermore, both compounds increased *p21Cip1/Waf1/Sdi1, p27Kip1*, and* p57Kip2* significantly. Additionally, zebularine and TSA decreased *DNMTs* and *HDACs *gene expression respectively.

**Conclusion:**

The zebularine and trichostatin A can reactivate the CIP/KIP family through inhibition of *DNMTs *and *HDACs *genes activity.

## Introduction

A pattern of epigenetic modifications and changes, DNA methylation and histone modification, is central to many human cancers. Posttranslational modifications affect chromatin structure and compaction resulting in changes in cell behavior and altered gene expression. Aberrant altered epigenomic patterns and gene expression are significant features of many cancers. Epigenetic modifications comprising DNA methylation, histone methylation, and histone acetylation plays an important role in the induction and progression of numerous cancers (Ellis et al., 2009). In fact, dysregulated epigenetic modifications play a significant role in driving cancer development and progression. A variety of tumor suppressor genes (TSGs) have been demonstrated to be silenced in several cancers. Indeed, the TSGs silencing is accompanied by histone deacetylation and DNA hypermethylation (Bachman et al., 2003). The mammalian somatic cell cycle is an ordered, tightly-regulated process with multiple checkpoints. The cycle is divided into four phases comprising initial growth (G1), DNA synthesis (S), a gap (G2), and mitosis (M). The molecular machinery of the cell cycle is a family of enzymes, the cyclin-dependent kinases (Cdks). The active form of these enzymes is a complex consisting of a kinase and a cyclin. Both cyclin and Cdk are positive regulators of cell cycle progression whereas, cyclin-dependent kinase inhibitors (CKIs) are negative regulators of cell cycle which stop or break the cycle. These negative inhibitors comprise two families including *INK* and *CIP/KIP* families. The *INK* family consists of *INK4A (p16), INK4B (p15), INK4C (p18),* and *INK4D (p19)*. The* CIP/KIP *family includes *CIP1 (p21), KIP1 (p27)*, and *KIP2 (p57)* (Park et al., 2003). Recent in vitro studies have shown that two known mechanisms of epigenetic alteration consisting, methylation and histone deacetylation seem to be the best candidate mechanisms for inactivation of* CIP/KIP* family in numerous cancers such as gastric cancer (Shin et al., 2000; Borges et al., 2010), lung cancer (Fang et al., 2002), hepatocellular carcinoma (HCC), pancreatic cancers, and acute myeloid leukemia (Kikuchi et al., 2002), and colon cancer (Colo-320 and SW1116) (Fang et al., 2004). There are several reports regarding the association between overexpression of DNA methyltransferases 1, 2 and 3 and DNA methylation in various cancers comprising gastric cancer (Ding et al., 2008), colon cancer *HCT116, LS180, HT29/219, Caco2*, and *SW742* cell lines (Sarabi et al., 2015). Furthermore, the overexpression of *class I* and *II HDAC* has been reported in colon cancer (Mariadason., 2008). Silenced TSGs are obvious targets for reactivation by DNA methyltransferase inhibitors (*DNMTIs*) such as 5-aza-2′-deoxycytidine (5-Aza-CdR), 5-azacytidine (5-Aza-CR) and zebularine. Numerous investigations have indicated that zebularine, a general inhibitor of DNA methylation, inhibits *DNMTs* in human breast cancer *MDA-MB-231 *and *MCF-7* (Billam et al., 2010), *T24* bladder carcinoma (Ben-Kasus et al., 2005), colon cancer *SW480* cells, *HT29, SW48* and *HCT116 *(Ikehata et al., 2014). Besides, it has been identified that histone deacetylase inhibitors (*HDACIs*) can suppress* class I* and* II* *HDAC* in colon cancer (Mariadason., 2008). Previously, we evaluated the effect of trichostatin A (TSA) on HCC (Sanaei et al., 2017; Sanaei et al., 2018; Leu et al., 2003) and also the effect of DNA demethylating agent* 5-AZA-CdR* on this cancer (Sanaei et al., 2019). The present study was designed to investigate the effect of zebularine and trichostatin A on *p21Cip1/Waf1/Sdi1, p27Kip1, p57Kip2, DNMT1, DNMT3a *and* DNMT3b, Class I HDACs (HDACs 1, 2, 3)* and *Class II HDACs (HDACs 4, 5, 6)* gene expression, cell growth inhibition and apoptosis induction in colon cancer *LS 174T* cell line. 

## Materials and Methods

*Materials*

The human colon cancer LS 174T cell line was kindly provided from the National Cell Bank of Iran-Pasteur Institute and maintained in Dulbecco’s modified Eagle’s medium (DMEM) supplemented with antibiotics and fetal bovine serum 10% in a humidified atmosphere of 5% CO_2_ in air at 37^o^C. All reagents (zebularine and TSA), kits, and instruments used in RNA extraction, reverse transcription, and real-time PCR were provided as obtained previously (Sanaei M et al., 2017; Sanaei M et al., 2018; Sanaei M et al., 2019).

*Cell culture and cell viability*

The colon cancer LS 174T cell viability was measured using 3 (4,5 dimethyl 2 thiazolyl) 2, 5 diphenyl 2H tetrazolium bromide (MTT) assay. The LS 174T cells were cultured and seeded in 96-well plates at a density of 4 × 10^5^ cells per well and treated with zebularine (0, 10, 25, 50, 100, and 200 μM) and TSA (0, 1, 5, 10, 25, and 50 μM) for 24 and 48 h. The control groups were treated with Dimethyl sulfoxide (DMSO), 0.05 %, only. After incubation for 24 and 48 h, the LS 174T cells were exposed to MTT, MTT (0.5 mg/mL) was added to each well for 4 h. Finally, the MTT metabolite, the blue MTT formazan, was dissolved in DMSO (200 mL) and the optical density was detected by a microplate reader at a wavelength of 570 nM. Each experiment was repeated three times (triplicates).

*Cell apoptosis assay*

To determine the apoptotic LS 174T cells, the cells were treated with cultured and seeded at a density of 4 × 10^5^ cells/well and treated with zebularine (50 μM) and TSA (5 μM) for 24 and 48 h. Following treatment, the cells were harvested by trypsin and washed twice with PBS and then resuspended in Binding buffer (1x). Subsequently, the cells were stained with annexin V-FITC (5 μl) and propidium iodide (5 μl) in the dark at room temperature for 15 min. Finally, the LS 174T apoptotic cells were counted by FACScan flow cytometry (Becton Dickinson, Heidelberg, Germany).

*Real-time Quantitative Reverse Transcription PolymeraseChain Reaction (qRT-PCR)*

The qRT-PCR was performed to determine the relative expression level of *p21Cip1/Waf1/Sdi1, p27Kip1, p57Kip2, DNMT1, DNMT3a, DNMT3b, Class I HDACs *(*HDACs 1, 2, 3*) and *Class II HDACs (HDACs 4, 5, 6) *genes. The colon cancer LS 174T cells were cultured at a density of 4 × 10^5^ cells/well and treated with zebularine (50 μM) and TSA (5 μM), as alone and combined, for 24 and 48 h, based on IC5o values. After 24 and 48 h of incubation, the total RNA was harvested using the RNeasy kit (Qiagen, Valencia, CA) according to the manufacturer protocol and treated by RNasefree DNase (Qiagen). The RNA was transcribed to complementary DNA (cDNA). QRT-PCR was done to assess the expression level of the mentioned genes as described previously (Sanaei et al., 2019). The program for the PCR was as we reported previously (Sanaei et al., 2019). The primer sequences of the genes are indicated in table1. GAPDH was used as an endogenous control. Data were analyzed using the comparative Ct (ΔΔct) method. 

## Results

*Result of cell viability by the MTT assay*

**Figure 1:**
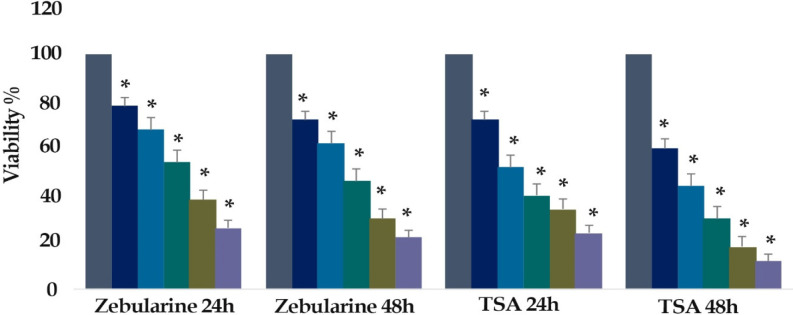
In Vitro Effects of Zebularine (0, 10, 25, 50, 100, and 200 μM) and TSA (0, 1, 5, 10, 25, and 50 μM) on LS 174T Cells Viability Determined by MTT Assay at 24 and 48 h. As shown, from right to the left, the first column of each group belongs to the control group. Values are means of three experiments in triplicate. Asterisks (*) demonstrate significant differences between treated and untreated control groups

To test the effect of zebularine (0, 10, 25, 50, 100, and 200 μM) and TSA (0, 1, 5, 10, 25, and 50 μM) on the colon cancer LS 174T cell viability, MTT assay was utilized. Our findings showed that the rate of cell growth inhibition was significantly increased in than that in control groups after 24 and 48 h. Results showed that the number of viable LS 174T cells decreased significantly, as the concentration of the compounds and duration increased; indicating a dose- and duration-dependent relationship (P< 0.001), Figure 1. The IC_50_ values were determined with approximately 50 and 5 μM for zebularine and TSA respectively. 

*Result of cell apoptosis assay*

**Figure 2:**
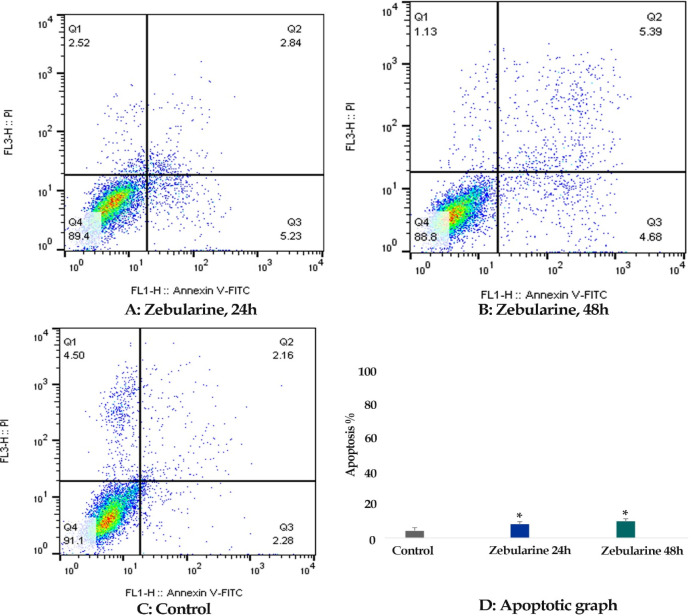
The Apoptotic Effect of Zebularine (50 μM) on Colon Cancer LS 174T Line Cell versus Control Groups at Different Periods (24 and 48h). The cells were treated with this zebularine for 24 and 48h and then the apoptotic effect was evaluated by flow cytometric analysis. Results were obtained from three independent experiments and were expressed as mean ± standard error of the mean

**Figure 3:**
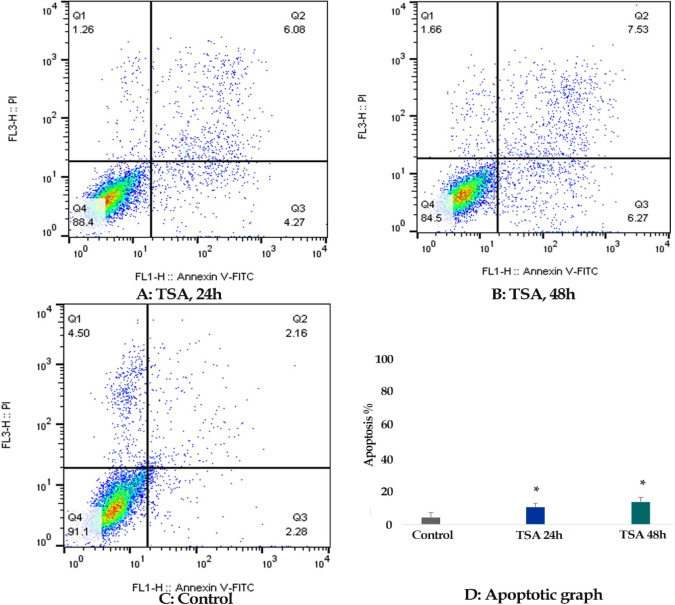
The Apoptotic Effect of TSA (5 μM) on Colon Cancer LS 174T Line Cell versus Control Groups at Different Periods (24 and 48h). The cells were treated with this zebularine for 24 and 48h and then the apoptotic effect was evaluated by flow cytometric analysis. Results were obtained from three independent experiments and were expressed as mean ± standard error of the mean

**Figure 4:**
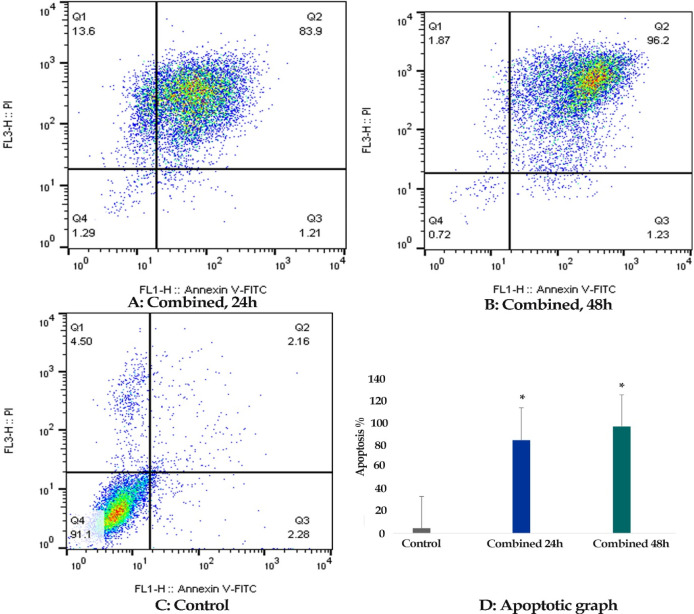
The Apoptotic Effect of Zebularine (50 μM) in Combination with TSA (5 μM) on LS 180 Cell versus Control Groups at Different Periods (24 and 48h). The cells were treated with combined agents for 24 and 48h and then the apoptotic effect was evaluated by flow cytometric analysis. Results were obtained from three independent experiments and were expressed as mean ± standard error of the mean

**Figure 5.**
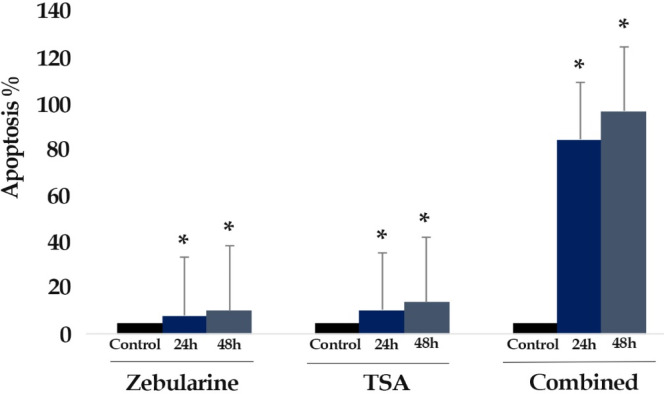
The Comparative Apoptotic Effects of Zebularine (50 μM) in Comparison to and in Combination with TSA (5 μM) on LS 174T Cells. Asterisks (*) indicate significant differences between the treated and untreated control groups. As demonstrated above, TSA had a more significant apoptotic effect on LS 174T cells in comparison to zebularine. The combined treatment had a maximum effect on apoptosis

Flow cytometric analysis was achieved to determine whether zebularine (50 μM) and TSA (5 μM) can induce apoptosis in colon cancer LS 174T line. The percentage of treated and un-treated LS 174T apoptotic cells was evaluated by staining with annexin V-FITC and PI after 24 and 48 h of treatment. After treatment with zebularine and TSA, as alone and combined, the apoptosis percentage increased significantly as shown in Table 2 and Figures 2-4. Both compounds had a time-dependent manner. The apoptotic effect of TSA was stronger than that of zebularine. Further, maximum apoptosis was seen in the groups treated with combined agents, Figure 5.

*Result of determination of genes expression*

**Figure 6:**
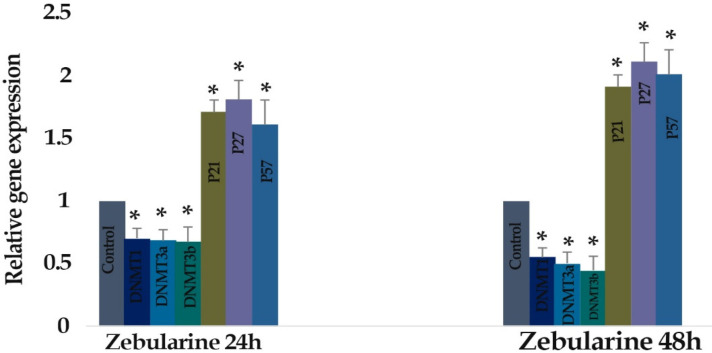
The Relative Expression Level of DNA Methyltransferases (DNMT1, 3a, and 3b), *p21Cip1/Waf1/Sdi1, p27Kip1*, and* p57Kip2* in the *LS 174T* Cell Line Treated with Zebularine (50 μM) versus Untreated Control Groups at Different Periods (24 and 48h). The first column of each group belongs to the untreated control group and the others belong to the treated cells with zebularine. Asterisks (*) indicate significant differences between the treated and untreated groups

**Figure 7.**
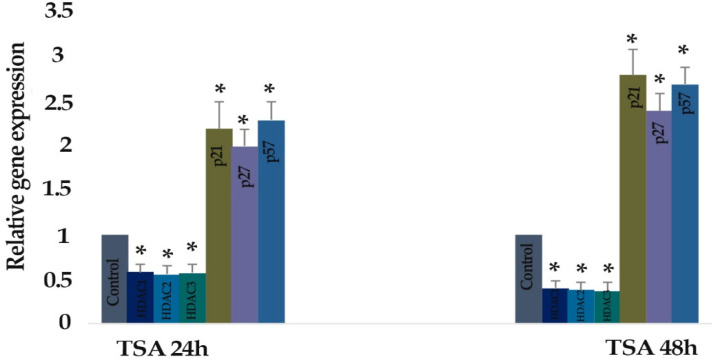
The Relative Expression Level of *Class I HDACs (HDACs 1, 2, 3)*, *Class II HDACs (HDACs 4, 5, 6), p21Cip1/Waf1/Sdi1, p27Kip1,* and *p57Kip2* Gene in the *LS 174T *Cell Line Treated with TSA (5 μM) versus Untreated Control Groups at Different Periods (24 and 48h). The first column of each group belongs to the untreated control group and the others belong to the treated cells with TSA. Asterisks (*) indicate significant differences between the treated and untreated groups

**Figure 8:**
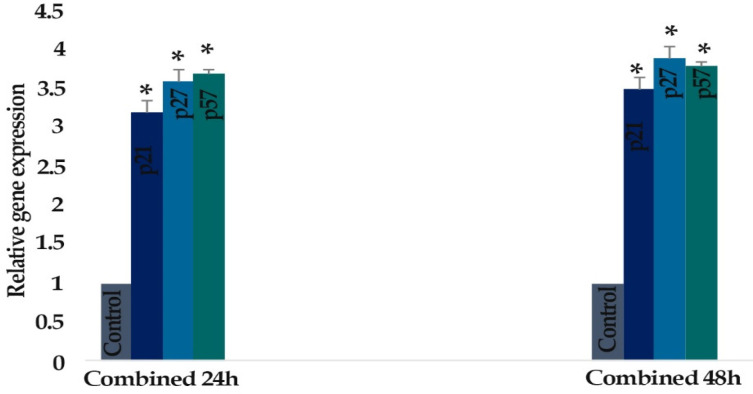
The Relative Expression Level of *p21Cip1/Waf1/Sdi1, p27Kip1,* and *p57Kip2* in the* LS 174T *Cell Line Treated with Zebularine (50 μM) in Combination with TSA (5 μM) versus Untreated Control Groups at Different Periods (24 and 48h). The first column of each group belongs to the untreated control group and the others belong to the treated cells with the combined components. Asterisks (*) indicate significant differences between the treated and untreated groups

**Figure 9:**
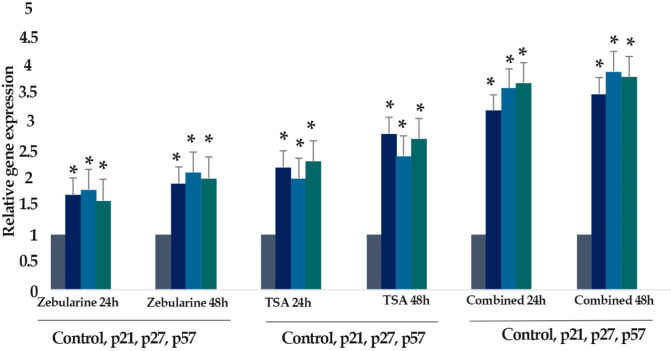
The Relative Expression Level of *p21Cip1/Waf1/Sdi1, p27Kip1*, and *p57Kip2* in the *LS 174T* Cell Line Treated with Zebularine (50 μM) and TSA (5 μM), Individually and Combined, versus Untreated Control Groups at Different Periods (24 and 48h). The first column of each group belongs to the untreated control group and the others belong to the treated cells with the zebularine and TSA. Asterisks (*) indicate significant differences between the treated and untreated groups

The effect of zebularine (50 μM) and TSA (5 μM) on the *p21Cip1/Waf1/Sdi1, p27Kip1, p57Kip2, DNMT1, DNMT3a, DNMT3b, Class I HDACs (HDACs 1, 2, 3) *and *Class II HDACs (HDACs 4, 5, 6)* gene expression was investigated by quantitative real-time RT-PCR analysis. The result indicated that treatment of LS 174T cells with zebularine (50 μM) and TSA (5 μM) for 24 and 48 h reactivated the *p21Cip1/Waf1/Sdi1, p27Kip1, p57Kip2 *gene, down-regulated *DNMT1, DNMT3a, DNMT3b, Class I HDACs (HDACs 1, 2, 3)* and *Class II HDACs (HDACs 4, 5, 6)* gene expression. The *CIP/KIP* family *(p21Cip1/Waf1/Sdi1, p27Kip1, p57Kip2*) genes were more sensitive to TSA compared with zebularine. It means that TSA had a more significant effect on the up-regulation of* p21Cip1/Waf1/Sdi1, p27Kip1, p57Kip2*, Figures 6-9.

## Discussion

The mammalian cell cycle is regulated via interactions between cyclins and a family of negative cell cycle regulators classified into two families, the *CIP/KIP *family, and the* INK4* family. DNA methylation patterns and histone deacetylation of these families strongly correlate with tumorigenesis (Tsou et al., 2002). DNA methylation is catalyzed by *DNMT1, DNMT3a*, and *DNMT3b*. Furthermore, histone deacetylation is catalyzed by *HDACs*. Therefore, *DNMTs* and *HDACs* induce hypermethylation and histone deacetylation respectively resulting in TSGs transcriptional repression and cancer induction (Li et al., 2007). The DNMTIs and HDACIs can reactivate epigenetically silenced TSGs (Yan et al., 2015). In the present study, zebularine and TSA reactivated the *p21Cip1/Waf1/Sdi1, p27Kip1, p57Kip2 *gene, down-regulated *DNMT1, DNMT3a, DNMT3b, Class I HDACs (HDACs 1, 2, 3)* and *Class II HDACs (HDACs 4, 5, 6)* gene resulting in cell growth inhibition and apoptosis induction. The *CIP/KIP* family *(p21Cip1/Waf1/Sdi1, p27Kip1, p57Kip2)* gene was more sensitive to TSA compared with zebularine. It means that TSA had a more significant effect on the up-regulation of *p21Cip1/Waf1/Sdi1, p27Kip1, p57Kip2.* In consistent with our result, it has been reported that zebularine can restore *p21Cip1/Waf1/Sdi1 *in colon cancer *SW48* and *HT-29* (Flis et al., 2014), *CT15* and *HT-29* colon cancer (Cheng et al., 2004). The other members of the *DNMTI *family act by the same pathway as reported for 5-azacitidine. It reactivates* p21Cip1/Waf1/Sdi1in HCT 116* colon cancer cells (Jiemjit et al., 2008),* p27Kip1* in colon cancer *SW1116* cells (Lu et al., 2007), *Caco2, RKO, SW48, SW480, Colo201, Colo205, Colo320, BM314, DLD-1*, and *HT29* (Kikuchi et al 2002). Further, we evaluated the molecular mechanism through which zebularine reactivate *p21Cip1/Waf1/Sdi1, p27Kip1, p57Kip2 *gene and found that this agent targets some of *DNMTIs (DNMT1, DNMT3a,* and *DNMT3b*). A similar mechanism (*DNMT* inhibition) has been demonstrated in colon cancer *HCT116* cells (Yang et al., 2013), HCC *Hep G2 *(Nakamura et al., 2013), cholangiocarcinoma cell lines TFK-1 and HuCCT1 (Nakamura et al., 2015). After assessment of the effect of *TSA on p21Cip1/Waf1/Sdi1, p27Kip1, p57Kip2* gene expression, we further determined the effect of TSA on Class *I HDACs (HDACs 1, 2, 3) *and *Class II HDACs (HDACs 4, 5, 6)* gene expression. As we reported, this agent down-regulated both* Class I *and *II HDACs*. Similarly, it has been shown that TSA down-regulates* class I* and* II HDACs* in colon cancer cells *SW620, SW480*, and *DLD1* (Krishnan et al., 2010), *class I *and *II HDACs* in human prostate cancer *LNCaP *cells (Thakur et al., 2011). Further molecular mechanisms of TSA including down-regulation of *HDAC8* in colon cancer cell line *SW620* (Hu et al., 2003), *HDACs 5* and *8 *in lung cancer, breast cancer, and skin cancer cells (Chang et al., 2012). As mentioned above, we only evaluated the effect of TSA on *Class I *and *II HDACs* in colon cancer. Therefore, the investigation of TSA on the other classes of *HDACs *is recommended.

In summary, our findings indicated that zebularine and TSA can down-regulate *DNMT1, DNMT3a, DNMT3b, *class *I HDACs (HDACs 1, 2, 3)* and *class II HDACs (HDACs 4, 5, 6)* resulting in reactivation of *p21Cip1/Waf1/Sdi1, p27Kip1, p57Kip2,* cell growth inhibition and apoptosis induction of colon cancer* LS 174T* cell line. 

**Table 1:** The Primer Sequences of *p21Cip1/Waf1/Sdi1*, *p27Kip1, p57Kip2, DNMT1, DNMT3a, DNMT3b, Class I* *HDACs (HDACs 1, 2, 3) *and *Class II HDACs (HDACs 4, 5, 6)*

Primer Name	Primer Sequences (5' to 3')	References
DNMT1 Forward	GAG GAA GCT GCT AAG GAC TAG TTC	Sanaei et al., 2018
DNMT1 Reverse	ACT CCA CAA TTT GAT CAC TAA ATC	Sanaei et al., 2018
DNMT3a Forward	GGA GGC TGA GAA GAA AGC CAA GGT	Sanaei et al., 2018
DNMT3a Reverse	TTT GCC GTC TCC GAA CCA CAT GAC	Sanaei et al., 2018
P21 Forward	AGG CGC CAT GTC AGA ACC GGC TGG	Sanaei et al., 2019
P21 Reverse	GGA AGG TAG AGC TTG GGC AGG C	Sanaei et al., 2019
P 27 Forward	ATG TCA AAC GTG CGA GTG TCT AAC	Sanaei et al., 2019
P 27 Reverse	TTA CGT TTG ACG TCT TCT GAG GCC A	Sanaei et al., 2019
P 57 Forward	GCGGCGATCAAGAAGCTGTC	Sanaei et al., 2019
P 57 Reverse	CCGGTTGCTGCTACATGAAC	Sanaei et al., 2019
DNMT3b Forward	TAC ACA GAC GTG TCC AAC ATG GGC	Leu et al., 2003
DNMT3b Reverse	GGA TGC CTT CAG GAA TCA CAC CTC	Leu et al., 2003
GAPDH Forward	TCCCATCACCATCTTCCA	Jin et al., 2008
GAPDH Reverse	CATCACGCCACAGTTTCC	Jin et al., 2008
HDAC1 Forward	GACACGCCAAGTGTGTGGAA	Song et al., 2015
HDAC1 Reverse	CCTCCCAGCATCAGCATAGG	Song et al., 2015
HDAC2 Forward	ACATGAGCAATGCGGAGAAAT	Song et al., 2015
HDAC2 Reverse	TCTGCCATCTTGTGGTACAGTGA	Song et al., 2015
HDAC3 Forward	CCTTTTCCAGCCGGTTATCA	Song et al., 2015
HDAC3 Reverse	ACAATGCACGTGGGTTGGT	Song et al., 2015
HDAC4 Forward	TCAGATCGCCAACACATTCG	Song et al., 2015
HDAC4 Reverse	ACGGGAGCGGTTCTGTTAGA	Song et al., 2015
HDAC5 Forward	CCATTGGAGACGTGGAGTACCT	Song et al., 2015
HDAC5 Reverse	GCGGAGACTAGGACCACATCA	Song et al., 2015
HDAC6 Forward	TCGCTGCGTGTCCTTTCAG	Song et al., 2015
HDAC6 Reverse	GCTGTGAACCAACATCAGCTCTT	Song et al., 2015

**Table 2:** Percentage of Apoptosis in the Groups Treated with Zebularine and TSA, as Alone and Combined, at Different Periods

Drug	Dose/ μM	Duration/ h	Apoptosis %	P-value
Zebularine	50	24	8.07	P < 0.001
	50	48	10.07	P < 0.001
TSA	5	24	10.35	P < 0.001
	5	48	13.8	P < 0.001
Zebularine/TSA	50/5	24	85.11	P < 0.001
	50/5	48	97.43	P < 0.001

**Table 3:** The Relative Expression Level of *DNMTs (DNMT1, DNMT3a, *and* DNMT3b*), *CIP/KIP* Family (*p21Cip1/Waf1/Sdi1, p27Kip1*, and *p57Kip2*) *HDAC1, HDAC2*, and *HDAC3* Genes

Cell line	Gene	Drug	Dose (μM)	Duration (h)	Expression	*P*-value
LS 174T	*DNMT1*	Zebularine	50	24	0.7	0.023
LS 174T	*DNMT1*	Zebularine	50	48	0.55	0.001
LS 174T	*DNMT3a*	Zebularine	50	24	0.68	0.014
LS 174T	*DNMT3a*	Zebularine	50	48	0.5	0.001
LS 174T	*DNMT3b*	Zebularine	50	24	0.67	0.011
LS 174T	*DNMT3b*	Zebularine	50	48	0.44	0.01
LS 174T	*P21*	Zebularine	50	24	1.7	0.004
LS 174T	*P21*	Zebularine	50	48	1.9	0.001
LS 174T	*P27*	Zebularine	50	24	1.8	0.001
LS 174T	*P27*	Zebularine	50	48	2.1	0.001
LS 174T	*P57*	Zebularine	50	24	1.6	0.001
LS 174T	*P57*	Zebularine	50	48	2	0.001
LS 174T	*HDAC1*	Trichostatin A	5 μM	24	0.58	0.001
LS 174T	*HDAC1*	Trichostatin A	5 μM	48	0.4	0.001
LS 174T	*HDAC2*	Trichostatin A	5 μM	24	0.56	0.002
LS 174T	*HDAC2*	Trichostatin A	5 μM	48	0.38	0.001
LS 174T	*HDAC3*	Trichostatin A	5 μM	24	0.57	0.001
LS 174T	*HDAC3*	Trichostatin A	5 μM	48	0.37	0.001
LS 174T	*P21*	Trichostatin A	5 μM	24	2.2	0.001
LS 174T	*P21*	Trichostatin A	5 μM	48	2.8	0.001
LS 174T	*P27*	Trichostatin A	5 μM	24	2	0.001
LS 174T	*P27*	Trichostatin A	5 μM	48	2.4	0.001
LS 174T	*P57*	Trichostatin A	5 μM	24	2.3	0.001
LS 174T	*P57*	Trichostatin A	5 μM	48	2.7	0.001

**Table 4:** The Relative Expression Level of p21Cip1/Waf1/Sdi1, p27Kip1, p57Kip2 Genes in the Groups Treated with Combined Treatment

Cell line	Gene	Drug	Dose (μM)	Duration (h)	Expression	*P*-value
LS 180	*P21*	Zebularine/TSA	25/5 μM	24	3.2	0.001
LS 180	*P21*	Zebularine/TSA	25/5 μM	48	3.5	0.001
LS 180	*P27*	Zebularine/TSA	25/5 μM	24	3.6	0.001
LS 180	*P27*	Zebularine/TSA	25/5 μM	48	3.9	0.001
LS 180	*P57*	Zebularine/TSA	25/5 μM	24	3.7	0.001
LS 180	*P57*	Zebularine/TSA	25/5 μM	48	3.8	0.001
